# Viruses from Nonhuman Primates

**DOI:** 10.3201/eid1211.060659

**Published:** 2006-11

**Authors:** Regis A. Vilchez

**Affiliations:** * Boehringer Ingelheim Pharmaceuticals, Inc., Ridgefield, Connecticut, USA

**Keywords:** SV40, polyomaviruses, rhesus macaques, letter

To the Editor: I read with interest the article by Jones-Engel et al. (*1*), which described the frequency of viruses infecting temple rhesus macaques. The investigation included the polyomavirus simian virus 40 (SV40), a pathogen recognized to have infected millions of humans who were vaccinated with polio vaccines produced in cultures of rhesus monkey kidney cells (*2*,*3*). The authors indicated that technologic advances have improved the specificity of detecting SV40 antibodies and used an enzyme immunoassay based on viruslike particles (VLPs) to perform the analysis (*1*). However, the specificity of the SV40 enzyme immunoassay is problematic because studies with serum samples from macaques have found that antibodies are cross-reactive with polyomaviruses JCV and BKV (*4*). In addition, in monkey sera SV40 VLPs correlated with BKV antibodies. Similar conflicting results have been found in human studies that used polyomavirus VLPs assays (*3*). These limitations are the result of polyomavirus VLPs assays using expression of the VP1 capsid protein (*4*), a highly homologous gene among JCV, BKV, and SV40 (*3*). In contrast, modern molecular biology assays are the preferred method for the analysis of SV40 infections (*2*,*3*). In addition, these sensitive and specific techniques can provide insights into the distribution of SV40 strains and variants (*2*,*3*). This is important because recent data suggest that the biological properties of SV40 strains vary in vivo (*5*).

Because current evidence shows that SV40 infections are identified in some humans and that the virus is associated with selected human malignancies (*2*,*3*), prospective longitudinal studies that use molecular techniques are needed to examine the prevalence and ecology of SV40. The Institute of Medicine recognizes that the biologic evidence indicates that infections with this DNA virus could lead to cancer in humans and recommends targeted biologic research of SV40 in human populations (*2*).
