# Supplementary material for: Viruses from Nonhuman Primates
Source: Emerg Infect Dis. 2006 Nov;12(11):1802–3. doi: 10.3201/eid1211.060659 (PMC3394671; doi:10.3201/eid1211.060659)
Supplement: Related texts

# Reply

**Authors:** Lisa Jones-Engel, Raphael Viscidi

**Affiliations:** * University of Washington, Seattle, Washington, USA;; † Johns Hopkins University, Baltimore, Maryland, USA

**In response:** Dr. Vilchez (*1*) raised questions regarding the specificity of the SV40 viruslike particle (VLP)–based ELISA used to detect SV40 infection in temple monkeys (*2*). Although it is true that SV40 infection can elicit low-level, cross-reactive antibodies that recognize polyomavirus BKV and to a lesser extent polyomavirus JCV VLPs and, conversely, BKV and JCV infection may elicit low-titer SV40 cross-reactive antibodies (*3*), these antibodies do not pose a problem for serologic diagnosis of infection in natural hosts of these polyomaviruses. Unless one were to hypothesize that BKV and JCV could infect macaques, SV40 VLP-reactive antibodies could not possibly be produced by anything other than an SV40 infection. In addition, SV40 seroreactivity in macaques is generally very strong but cross-reactive responses are weak (*3*).

Specificity of SV40 seroreactivity in macaques has recently been demonstrated by competitive inhibition assays (*4*). SV40 reactivity was blocked by incubation of sera with SV40 VLPs but not significantly reduced by incubation with BKV or JCV VLPs. Specificity of BKV and JCV VLP reactivity in human sera has also been demonstrated by preabsorption with VLPs (*5*) and competitive inhibition assays (*4*). Thus, BKV VLP seroreactivity can be completely inhibited by BKV VLPs but not by JCV VLPs and vice versa. When polyomavirus VLP ELISAs are used to diagnosis cross-species infection, such as SV40 infection in humans, competitive inhibition assays are necessary to verify specificity of the response. Engels et al. (*6*), using SV40 VLP serology and competitive inhibition assays, recently reported evidence for possible infection of zoo workers with SV40. We agree with Dr. Vilchez that molecular biology assays, such as PCR, play a valuable role in viral diagnosis. However, these assays also have limitations in terms of sensitivity and specificity and therefore are best combined with the full range of viral diagnostic techniques to confirm infection. Serologic testing is more suitable than other assays for estimating cumulative infection.

## References

[1] Vilchez RA. Viruses from nonhuman primates [letter]. Emerg Infect Dis. 2006;12:1802–3.1728570710.3201/eid1211.060659PMC3394671

[2] Jones-Engel L, Engel GA, Heidrich J, Chalise M, Poudel N, Viscidi R, Temple monkeys and health implications of commensalism, Kathmandu, Nepal. Emerg Infect Dis. 2006;12:900–6.1670704410.3201/eid1206.060030PMC3373059

[3] Viscidi RP, Rollison DE, Viscidi E, Clayman B, Rubalcaba E, Daniel R, Serological cross-reactivities between antibodies to simian virus 40, BK virus, and JC virus assessed by virus-like-particle-based enzyme immunoassays. Clin Diagn Lab Immunol. 2003;10:278–85.1262645510.1128/CDLI.10.2.278-285.2003PMC150538

[4] Viscidi RP, Clayman B. Serological cross reactivity between polyomavirus capsids. Adv Exp Med Biol. 2006;577:73–84. 10.1007/0-387-32957-9_516626028

[5] Carter JJ, Madeleine MM, Wipf GC, Garcea RL, Pipkin PA, Minor PD, Lack of serologic evidence for prevalent simian virus 40 infection in humans. J Natl Cancer Inst. 2003;95:1522–30.1455987410.1093/jnci/djg074

[6] Engels EA, Switzer WM, Heneine W, Viscidi RP. Serologic evidence for exposure to simian virus 40 in North American zoo workers. J Infect Dis. 2004;190:2065–9. 10.1086/42599715551203
